# Outcome of conservative treatments in patients with TMJ retrodiscal layer rupture or disc perforation

**DOI:** 10.1007/s00784-025-06177-9

**Published:** 2025-01-30

**Authors:** Mi-Sun Kong, Kyung-Hoe Huh, Hong-Seop Kho

**Affiliations:** 1 Department of Oral Medicine and Oral Diagnosis, School of Dentistry and Dental Research Institute, Seoul National University, 101, Daehak-ro, Jongno-gu, Seoul, 03080 South Korea; 2 Department of Oral and Maxillofacial Radiology, School of Dentistry and Dental Research Institute, Seoul National University, 101 Daehak-ro, Jongno-gu, Seoul, 03080 South Korea; 3 Institute on Aging, Seoul National University, Seoul, South Korea

**Keywords:** Conservative treatments, Disc perforation, Outcome, Retrodiscal layer rupture, Temporomandibular disorders, Temporomandibular joint

## Abstract

**Objectives:**

This study was aimed to investigate the efficacy of comprehensive conservative treatments in patients with temporomandibular joint (TMJ) retrodiscal layer rupture and/or disc perforation.

**Materials and methods:**

This was a retrospective study of thirty-one consecutive patients with findings of TMJ retrodiscal layer rupture and/or disc perforation using magnetic resonance imaging. Comprehensive stomatognathic system assessments were performed. Comprehensive treatment modalities were applied to each patient and treatment outcomes were analyzed. The changes in patient-reported symptoms and comfortable mouth opening (CMO) and maximum mouth opening (MMO) distances were analyzed between the baseline and after treatment.

**Results:**

Twenty-eight females and three males were included in this study. Four patients (12.9%) reported a history of facial injury and another four (12.9%) reported having rheumatoid arthritis. Unilateral chewing was the most frequently reported parafunctional habit, followed by clenching. The mean treatment duration was 24.3 ± 11.1 months. Most of the patients received more than one type of treatment. Both CMO and MMO distances increased significantly (*P* < 0.001) after treatment. Approximately three-quarters of patients reported partial improvement in symptoms, and one-fourth reported complete improvement.

**Conclusions:**

Comprehensive conservative treatments were effective and should be applied first in patients with TMJ retrodiscal layer rupture and/or disc perforation.

**Clinical relevance:**

Conservative treatments are recommended for patients with severe damage to the TMJ structures, such as retrodiscal layer rupture and/or disc perforation, before applying surgical approach.

**Supplementary Information:**

The online version contains supplementary material available at 10.1007/s00784-025-06177-9.

## Introduction

Temporomandibular disorders (TMD) encompass a group of musculoskeletal disorders that involve the temporomandibular joints (TMJs) and associated masticatory muscles. Patients with TMD present with pain in the temporomandibular area, limited mandibular function, and noise in the TMJ. Internal derangement (ID), degenerative joint disease (DJD), and arthralgia are common conditions associated with TMJ. Myalgia and myofascial pain are common conditions associated with the masticatory muscles. Headache and otologic symptoms are common associated complaints [1, 2].

The occurrence of TMJ DJD is usually accompanied by TMJ ID, especially anterior disc displacement (ADD) without reduction [1, 3]. Therefore, severe damage to the TMJ intra-articular tissue, usually resulting from repetitive overloading, could progress into “rupture of the retrodiscal layer” mostly at the junction between the disc and the posterior attachment rather than “perforation of the central part of the disc” [4–6]. Under the repetitive overloading, the extended ligament between the articular disc and the retrodiscal layer loses elasticity, deforms, thins, perforates, and ruptures [7]. Rupture in the retrodiscal layer may produce significant disc instability and the resulting increased intra-articular friction exacerbates degenerative process [8]. Both terminologies, “rupture of the retrodiscal layer” and “perforation of the disc”, have been used interchangeably and both conditions were broadly described as disc perforation in previous studies [4, 9].

Perforation of the articular disc in a broad sense has been reported in 5–15% of the patients with TMJ ADD in a series of autopsy materials [9]. Prevalence of disc perforation is higher in women and its incidence rate increases with age [9, 10]. Etiological factors involved include oral parafunctional habits, including bruxism [11], trauma history on the mandible [12], cervical spine and neck muscles-related diseases [11], and rheumatic diseases [13].

Because there are no pathognomonic symptoms associated with TMJ disc perforation, disc perforation is often found incidentally during diagnosis using advanced imaging techniques, especially in cases with severe changes in TMJs. Although TMJ arthrography or arthroscopy provides direct information regarding the integrity and position of the articular disc, their invasive nature limits their routine use [14, 15]. Thus, disc perforation of TMJs is usually detected via magnetic resonance imaging (MRI) with superior resolution in soft tissues [16–18].

TMJ disc perforation is usually treated by open or arthroscopic surgery, including perforation site suture or fixation, open discectomy, arthroplasty, and arthroscopic lavage or lysis [15, 19, 20]. Surgical management has shown controversial treatment outcomes, and complications, including exacerbation of degenerative arthritis, ankylosis, and facial nerve injury, have been reported [11, 21]. However, to the best of our knowledge, there has been no study focused on the efficacy of conservative therapies for patients with TMJ retrodiscal layer rupture and/or disc perforation. The purpose of this study was to investigate the efficacy of comprehensive conservative therapy in patients with TMJ retrodiscal layer rupture and/or disc perforation.

## Materials and methods

### Participants

This was a retrospective study involving patients who visited the TMJ and Orofacial Pain Clinic, Department of Oral Medicine, Seoul National University Dental Hospital between 1 Jan. 2009 and 31 Dec. 2021. Among the patients who were diagnosed with TMD based on the Research Diagnostic Criteria for TMD (RDC/TMD) [22], those with “rupture of the retrodiscal layer” and/or “disc perforation” in TMJ-MRI were consecutively included in the study. The study was approved by the Institutional Review Board of Seoul National University School of Dentistry (S-D20230022). This study was performed in line with the principles of the Declaration of Helsinki. The Institutional Review Board also approved exemptions from acquiring informed consent from the patients.

### Inclusion and exclusion criteria

The number of patients evaluated by one oral medicine doctor (HSK) during the period was 7,249. The number of patients who underwent TMJ-MRI was 521. Patients were included if they met the following criteria: (1) an interpretation of TMJ retrodiscal layer rupture and/or disc perforation in MRI, and (2) a treatment follow-up period of more than six months. Patients were excluded if they had (1) congenital deformities or developmental abnormalities of the TMJ and/or history of their treatments; (2) history of orthognathic surgery; (3) history of open or arthroscopic surgery in TMJ; or (4) history of head and neck cancers or their treatments.

Thirty-six patients were identified as having TMJ retrodiscal layer rupture and/or disc perforation in MRI by an oral and maxillofacial radiologist (KHH). Two oral medicine doctors (MSK and HSK) independently reviewed the electronic medical records to identify potentially eligible patients meeting the inclusion/exclusion criteria and to collect and extract data. Disagreements between the two doctors were resolved through discussion. After screening, five patients with less than 6 months of follow-up were excluded, and there were no patients who met other exclusion criteria. Finally, thirty-one patients were selected.

### Clinical evaluation

All participants were thoroughly assessed the stomatognathic system including examination of mandibular movements, TMJ noise, TMJ and masticatory muscle palpations, and oral parafunctional habit-related findings based on the RDC/TMD. All patients were diagnosed as follows: (1) Group I: myofascial pain without limited opening and myofascial pain with limited opening (< 40 mm), (2) Group II: disc displacement with reduction, disc displacement without reduction with limited opening (≤ 35 mm), and disc displacement without reduction without limited opening (> 35 mm); and (3) Group III: arthralgia, osteoarthritis, and osteoarthrosis [22]. Evaluation was performed by one doctor (HSK).

### Radiographic and MRI evaluations

**Fig. 1:**
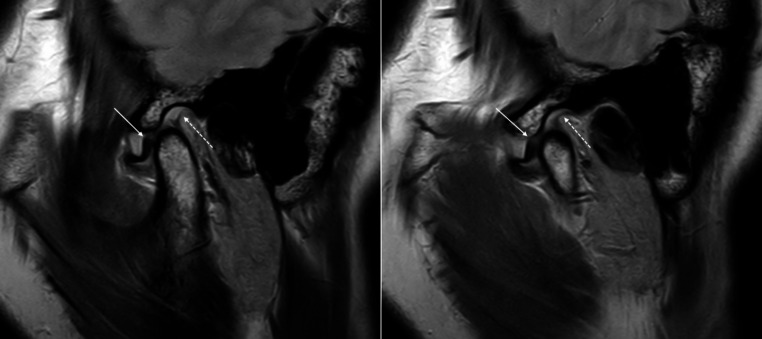
Two consecutive modified-sagittal proton density-weighted MR images with open mouth position, demonstrating retrodiscal layer rupture of the right temporomandibular joint (patient #14). Note the separation between the posterior band of the disc (arrow) and the retrodiscal layer (dashed arrow)

**Fig. 2:**
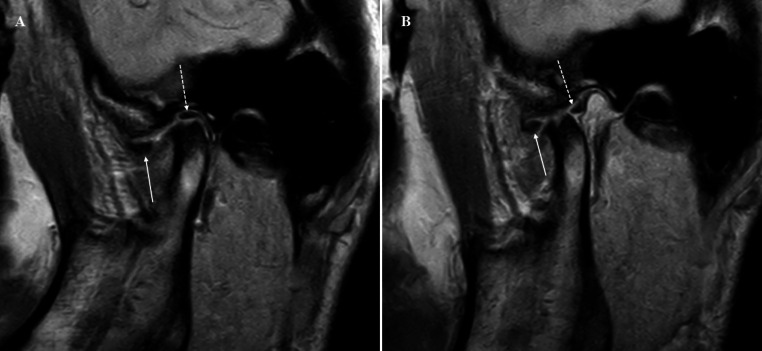
Modified-sagittal proton density-weighted MR images with closed mouth (**A**) and open mouth positions (**B**), representing disc perforation of the left temporomandibular joint (patient #15). Interruption in the intermediate zone of the disc with separation of the anterior (arrow) and posterior (dashed arrow) bands is noted

TMJ plain radiography, cone beam computed tomography (CBCT), and MRI were performed. TMJ plain radiography included panoramic and transcranial radiographs. The changes of the TMJ articular surface on modified-sagittal and -coronal CBCT images, adjusted to the axis of each condylar head, were evaluated to detect osseous changes, including surface erosion, subcortical defect, osteophyte, and generalized sclerosis. MRI was performed using a 3.0 T imager. Modified-sagittal and -coronal proton density-weighted MR images with a closed mouth position, modified-sagittal T2-weighted MR images with a closed mouth position, and proton density-weighted modified-sagittal MR images with an open mouth position were evaluated. When the continuity of the disc was interrupted in the intermediate zone with separated anterior and posterior bands in both the open and closed mouth positions, the patient was diagnosed as disc perforation. When the superior retrodiscal layer lost its union with the posterior band, the patient was diagnosed as rupture of retrodiscal layer [23]. The evaluation of radiographic and MR images was performed by an oral and maxillofacial radiologist (KHH) with 20 years of experience (Figs. 1 and 2).

### Evaluation of treatment outcome

Treatment modalities applied to the patients included behavioral therapy, physical therapy, including moist hot pack, ultrasound therapy, and exercise, medications, and oral stabilization splint therapy. An individualized treatment plan was made for each patient.

Regarding behavioral therapy, patients were counseled and educated on behavioral modifications that affect TMD symptoms. These included the elimination of parafunctional habits and avoidance of extreme mandibular movement, reduction of high psychological distress, and increase in sleep quality. Physical therapy included moist hot pack and ultrasound therapy on the affected side. Exercise therapy included tongue posture, hinge movement of the mandible, avoidance of protruding neck posture, and neck and shoulder exercises within the range of motion. The prescribed medications were nonsteroidal anti-inflammatory drugs (NSAIDs) and muscle relaxants. The oral stabilization occlusal splint was made of hard acrylic resin with even bilateral occlusal contact with the premolars and molars in the centric relation position and canine guidance. Recall check-ups were conducted almost monthly. Changes in symptoms and parameters of the interincisal distances of comfortable mouth opening (CMO) and maximum mouth opening (MMO), palpation of TMJ capsules and masticatory muscles, and noise were recorded.

Regarding treatment outcomes, changes in patient-reported symptoms and interincisal distances of CMO and MMO between the baseline and after treatment were analyzed. The changes in masticatory muscle pain and TMJ capsular pain were also analyzed between the baseline and after treatment. The changes in patient-reported symptoms were evaluated as follows: no improvement or worsening of symptoms, partial improvement of symptoms, and complete improvement of symptoms.

### Statistical analysis

For the treatment outcomes, the paired t-test or Wilcoxon signed rank test based on the normality test was used to compare CMO and MMO between the baseline and after treatment. Pearson’s chi-square test was used to analyze differences between the baseline and after treatment for the presence or absence of masticatory muscle pain and TMJ capsular pain. Statistical analysis was performed using SPSS 26.0 software (SPSS, Chicago, IL, USA). A *P* value less than 0.05 was considered statistically significant.

## Results

### Clinical characteristics

Table 1 and Supplementary Table 1 present the demographic and clinical characteristics of the patients with TMJ retrodiscal layer rupture and/or disc perforation. Twenty-eight females and three males, with a mean age of 50.4 ± 13.7 (20–78 years old) were included in this study. Six patients (one male and five females) were under the age of 40 and three (one male and two females) were in their 20s. Four patients (12.9%) reported a history of facial injuries caused by direct blows to the facial area, including the temporomandibular region. Four patients (12.9%) had rheumatoid arthritis. Among the six patients under the age of 40, two had rheumatoid arthritis and one had a history of facial injury. Regarding parafunctional habits, unilateral chewing (*n* = 20, 64.5%) was the most common, followed by clenching (*n* = 14, 45.2%). Twenty-six patients (83.9%) reported having at least one parafunctional habit.

**Table 1:** Demographic and clinical characteristics of patients with TMJ retrodiscal layer rupture and/or disc perforation; Mean ± SD, n (%)

Characteristics	*n* = 31
Age (year)	50.4 ± 13.7
Sex	
Male	3 (9.7)
Female	28 (90.3)
Rheumatoid arthritis	4 (12.9)
Trauma history on the facial area	4 (12.9)
Parafunctional habit	
Bruxism	3 (9.7)
Clenching	14 (45.2)
Bruxism and/or Clenching	14 (45.2)
Unilateral chewing	20 (64.5)
Chewing gum	3 (9.7)
Tongue thrusting	1 (3.2)
Patients with at least one parafunctional habit	26 (83.9)

TMJ, temporomandibular joint

### TMJ-MRI and TMJ-CBCT findings and TMD diagnosis

Table 2 and Supplementary Table 1 show the TMJ-MRI and TMJ-CBCT findings of the patients. Retrodiscal layer rupture (*n* = 24, 77.4%), disc perforation (*n* = 5, 16.1%), and combined type (*n* = 2, 6.5%) were observed on TMJ-MRI. Among sixty-two joints of thirty-one patients, thirty-six joints with retrodiscal layer rupture and/or disc perforation were observed. Unilateral involvement of retrodiscal layer rupture and/or disc perforation was observed in twenty-six patients (twenty-six joints), and bilateral involvement was observed in five patients (ten joints). Interestingly, all four patients with rheumatoid arthritis showed disc perforation, corresponding to four out of seven cases with disc perforation.

**Table 2:** TMJ-MRI and -CBCT findings and RDC/TMD Axis I diagnosis in patients with TMJ retrodiscal layer rupture and/or disc perforation; n (%)

	Number of joints	Number of patients
MRI findings		
Retrodiscal layer rupture	27 (75.0)	24^a^ (77.4)
Disc perforation	7 (19.4)	5^a^ (16.1)
Combined type	2 (5.6)	2^a^ (6.5)
Retrodiscal layer rupture and disc perforation		
Total	36	31
Retrodiscal layer rupture and/or disc perforation		
Unilateral involvement	26 (72.2)	26^b^ (83.9)
Disc displacement with reduction	0	0
Disc displacement without reduction	26 (72.2)	26 (83.9)
With degenerative joint disease	25 (69.4)	25 (80.6)
Without degenerative joint disease	1 (2.8)	1 (3.2)
With joint effusion	19 (52.8)	19 (61.3)
Without joint effusion	7 (19.4)	7 (22.6)
With muscle pain		17 (54.8)
Without muscle pain		9 (29.0)
Bilateral involvement	10 (27.8)	5^a^ (16.1)
Disc displacement with reduction	0	0
Disc displacement without reduction	10 (27.8)	5 (16.1)
With degenerative joint disease	10 (27.8)	5 (16.1)
Without degenerative joint disease	0	0
With joint effusion	3 (8.3)	3 (9.7)
Without joint effusion	7 (19.4)	5 (16.1)
With muscle pain		3 (9.7)
Without muscle pain		2 (6.5)
Total	36	31
Axis I diagnosis		
Group II and III		11 (35.5)
Group I, II, and III		20 (64.5)

CBCT, cone beam computed tomography; MRI, magnetic resonance imaging; RDC/TMD, Research Diagnostic Criteria for Temporomandibular Disorders; TMJ, temporomandibular joint

Axis I diagnosis: Group I: Myofascial pain with or without limited opening, Group II: Disc displacement with or without reduction, Group III: Other joint conditions (arthralgia, osteoarthritis, and osteoarthrosis)

CBCT was not taken in three patients. The diagnosis of degenerative joint diseases in three patients was based on plain radiography

^a^ One patient in the group did not take CBCT

^b^ Two patients in the group did not take CBCT

Table 2 and Supplementary Table 2 present the detailed TMD diagnoses. All thirty-six joints with retrodiscal layer rupture and/or disc perforation were diagnosed as disc displacement without reduction and all joints except for one showed degenerative changes. In fact, all patients had degenerative changes in their TMJs except for one patient (patient #14) in whom the degenerative changes were found in the opposite joint, not the joint with retrodiscal layer rupture. Joint effusion was observed in twenty-two joints. All effusion findings occurred in TMJs that showed retrodiscal layer rupture and/or disc perforation. Effusion findings were present in 19 of the patients with unilateral involvement and in 3 of the patients with bilateral involvement. There were no cases in which effusion findings were present in both TMJs. More than half of patients (*n* = 20, 64.5%) had masticatory muscle pain. All patients had multiple diagnoses based on the RDC/TMD; combined group II and III diagnoses were found in 11 patients (35.5%), whereas combined group I, II, and III diagnoses were found in 20 patients (64.5%).

### Evaluation of treatment outcomes

Table 3 and Supplementary Table 2 present the treatment types and durations. Duration of treatment was 24.3 ± 11.1 months. Various types of treatment were performed based on the patients’ symptoms. The most commonly applied treatment was the combination of physical therapy, NSAIDs, and oral stabilization splint therapy (*n* = 15, 48.4%), followed by the combination of physical therapy and oral stabilization splint therapy (*n* = 9, 29.0%).

**Table 3:** Type and duration of treatments for patients with TMJ retrodiscal layer rupture and/or disc perforation; Mean ± SD, n (%)

Treatment type and duration	
Total patients	*n* = 31
Treatment duration (months)	24.3 ± 11.1
Range	6–54
Types of treatment	
Physical therapy only	1 (3.2)
Physical therapy, NSAIDs	3 (9.7)
Physical therapy, stabilization splint therapy	9 (29.0)
Physical therapy, NSAIDs, stabilization splint therapy	15 (48.4)
Physical therapy, NSAIDs, muscle relaxants, stabilization splint therapy	3 (9.7)
Patients wearing stabilization splint	*n* = 27
Treatment duration (months)	18.7 ± 12.8
Range	1–48

NSAIDs, nonsteroidal anti-inflammatory drugs; TMJ, temporomandibular joint

Tables 4 and 5, and Supplementary Table 2 present the treatment outcomes. In all thirty-one patients, the interincisal distances of the CMO and MMO at the baseline were 32.1 ± 8.1 mm and 34.7 ± 7.2 mm, respectively. After treatment, the interincisal distances of the CMO and MMO were 39.3 ± 7.5 mm and 40.0 ± 7.2 mm, respectively, indicating a significant increase (*P* < 0.001 both in CMO and MMO). When comparing the baseline and after treatment, statistical significance was shown in myogenic pain (*P* = 0.005). Regarding patients’ reports of changes in symptoms, the percentages of patients who reported partial and complete improvement in symptoms were 74.2% (*n* = 23) and 25.8% (*n* = 8), respectively. There was no patient who reported no change or worsening of symptoms (Table 4).

**Table 4:** Treatment outcomes in the total number of patients (*n* = 31) with TMJ retrodiscal layer rupture and/or disc perforation; Mean ± SD, n (%)

Parameters	Baseline	After treatment	*P* value
CMO (mm)	32.1 ± 8.1	39.3 ± 7.5	< 0.001*
MMO (mm)	34.7 ± 7.2	40.0 ± 7.2	< 0.001*
Muscle pain			
With pain	20 (64.5)	9 (29.0)	0.005*
Without pain	11 (35.5)	22 (71.0)	
Capsular pain			
With pain	18 (58.1)	11 (35.5)	0.075
Without pain	13 (41.9)	20 (64.5)	
Treatment outcome			
No improvement or worsening of symptoms		0	
Partial improvement of symptoms		23 (74.2)	
Complete improvement of symptoms		8 (25.8)	

CMO, interincisal distance of comfortable mouth opening; MMO, interincisal distance of maximum mouth opening; TMJ, temporomandibular joint

Paired t-test, Wilcoxon signed rank test, or Pearson’s chi-square test was used to analyze differences in the parameters between the baseline and after treatment

* *P* < 0.05

**Table 5:** Treatment outcomes of patients wearing stabilization splint (*n* = 27), with TMJ retrodiscal layer rupture and/or disc perforation; Mean ± SD, n (%)

Parameters	Baseline	After treatment	*P* value
CMO (mm)	32.1 ± 8.4	40.0 ± 7.0	< 0.001*
MMO (mm)	34.9 ± 7.3	40.7 ± 6.6	< 0.001*
Muscle pain			
With pain	17 (63.0)	8 (29.6)	0.014*
Without pain	10 (37.0)	19 (70.4)	
Capsular pain			
With pain	16 (59.3)	9 (33.3)	0.056
Without pain	11 (40.7)	18 (66.7)	
Treatment outcome			
No improvement or worsening of symptoms		0	
Partial improvement of symptoms		21 (77.8)	
Complete improvement of symptoms		6 (22.2)	

CMO, interincisal distance of comfortable mouth opening; MMO, interincisal distance of maximum mouth opening; TMJ, temporomandibular joint

Paired t-test, Wilcoxon signed rank test, or Pearson’s chi-square test was used to analyze differences in the parameters between the baseline and after treatment

* *P* < 0.05

For the twenty-seven patients who received oral stabilization splint therapy, the mean interincisal distances of the CMO and MMO at the baseline were 32.1 ± 8.4 mm and 34.9 ± 7.3 mm, respectively. After treatment, the mean distances of the CMO and MMO increased significantly, reaching 40.0 ± 7.0 mm and 40.7 ± 6.6 mm, respectively (*P* < 0.001 both in CMO and MMO). When comparing the baseline and after treatment, statistical significance was also shown in myogenic pain (*P* = 0.014). Regarding the patients’ reports of changes in symptoms, the percentages of patients reporting partial and complete improvement of symptoms were 77.8% (*n* = 21) and 22.2% (*n* = 6), respectively (Table 5).

In the patients not wearing the splint (*n* = 4, patients #1, 8, 26, and 29), there were no significant differences in the interincisal distances of the CMO (*P* = 0.705) and MMO (*P* = 0.581), myogenic pain (*P* = 0.486), and capsular pain (*P* = 1) between the baseline and after treatment. The percentages of patients who reported partial and complete improvement of their symptoms were 50% (*n* = 2), and 50% (*n* = 2), respectively (Supplementary Table 3).

Three patients (#16, 18, and 26) were referred to the Department of Oral and Maxillofacial Surgery for additional surgical intervention. The patients received arthrocentesis treatment. Two (#16 and 26) of these patients reported improvement in their symptoms, but one patient (#18) complained of an increase in symptoms.

## Discussion

The purpose of this study was to investigate outcomes of comprehensive conservative treatments in patients with retrodiscal layer rupture and/or disc perforation. All patients showed significant increases in CMO and MMO distances and reported partial or complete improvement of in symptoms. This study showed that comprehensive conservative treatments were effective and should be applied first in patients with TMJ retrodiscal layer rupture and/or disc perforation before considering invasive surgical approaches.

In this study, most patients exhibiting retrodiscal layer rupture and/or disc perforation were women, and the mean age was approximately 50 years, consistent with those reported in previous studies [9, 10]. However, approximately 20% of the patients were under the age of 40 and half of them had rheumatoid arthritis or a history of facial injury. Macrotrauma to the face or microtrauma resulting from oral parafunctional habits have been the most frequently reported causes of TMJ overload and could cause retrodiscal layer rupture and/or disc perforation [12, 24]. Most patients in the present study reported oral parafunctional habits with high prevalence of unilateral chewing, bruxism, and/or clenching. These results were consistent with a previous study reporting that half of the patients with disc perforation had a history of bruxism [11]. Therefore, detailed history taking and evaluation, including facial and/or neck trauma and oral parafunctions, and their management are essential for patients with retrodiscal layer rupture and/or disc perforation.

Rheumatoid arthritis, one of the primary inflammatory joint diseases, is known to affect TMJs in between 19 and 88% of patients [13, 25–27]. Patients with TMD caused by rheumatoid arthritis show severe joint damage at a much faster rate than those resulting from degeneration with or without associated inflammation [28]. Therefore, it can be assumed that patients with rheumatoid arthritis who also have TMJ ID and oral parafunctional habits will experience more severe damage to joint structures. In the present study, all four patients with rheumatoid arthritis showed disc perforation, and all affected joints had disc displacement without reduction. Therefore, the possibility of primary inflammatory joint diseases, including rheumatoid arthritis, should be taken into consideration when evaluating patients with TMD showing severe joint damage.

In the present study, disc displacement without reduction was found in all affected joints, suggesting that an abnormal relationship of the intra-articular disc with the condyle and temporal bone is a risky environment for the development of retrodiscal layer rupture and/or disc perforation [4, 5, 29]. Considering the close relationship between disc displacement without reduction and degenerative changes in TMJ [1, 3], it was not surprising that degenerative changes were found in all but one of the thirty-six joints included in this study. A previous study also showed that, in most cases, retrodiscal layer rupture or articular disc perforation was accompanied by disc derangement and/or degenerative changes [30]. More than half of the patients with retrodiscal layer rupture and/or disc perforation showed masticatory muscle pain with or without TMJ capsular pain in the present study. Masticatory muscle pain in patients with TMD is reportedly closely associated with oral parafunctions and microtrauma [24, 31]. Comprehensive conservative treatments showed improvement in not only joint pain but also muscle pain in patients with retrodiscal layer rupture and/or disc perforation. Therefore, additional research is needed to determine the impact of improving muscle pain, which is not the focus of surgical treatments, on improving overall symptoms and quality of life in patients with severe joint damage.

The results of the present study showed that all patients with severe joint damage reported partial or complete improvement following comprehensive conservative treatments. Clinical features, including the mean interincisal distances of both CMO and MMO, increased significantly after treatments compared with the baseline in both groups, the total patients and the splint-wearing group. A decrease in masticatory muscle pain was also observed in patients who received comprehensive conservative treatment with splint therapy. Considering that the treatment outcomes were not statistically significant for the distances of both CMO and MMO in the four patients who did not receive splint therapy, the importance of the splint therapy could be further emphasized. However, the small number of patients who did not wear the splint could be a limitation in data interpretation. Comprehensive conservative treatments aim at decreasing the functional overload on the TMJs, inducing joint remodeling, rehabilitating masticatory muscles, recognizing or modifying contributing factors such as oral parafunction habits, and providing appropriate behavioral and psychologic support. All of these mechanisms were thought to have increased the interincisal distances of mouth opening and reduced TMJ capsular and masticatory muscle pain, as shown in the study results.

In this study, patients with retrodiscal layer rupture and/or disc perforation reported partial or complete improvement after treatment without any complications. Most previous studies focused on the therapeutic efficacy of surgery in patients with disc perforations [15, 19, 20]. Surgical approaches have posed some complications, including remaining muscle soreness, secondary degenerative arthritis, and ankylosis [32, 33]. Our results indicated that the comprehensive conservative treatments were effective and should be applied first in patients with TMJ retrodiscal layer rupture and/or disc perforation before considering invasive surgical approaches.

There were limitations in this study. First, given that this was a retrospective study, the results could not show the detailed degree of therapeutic efficacy of the comprehensive conservative treatments. The lack of better pain monitoring measures, such as visual analogue scales or algometer thresholds, limited more accurate assessment of treatment effects. Second, the effectiveness of each type of treatment could not be evaluated because treatments were performed in a comprehensive manner. Third, the treatment period, including the wearing period of stabilization splint, and the evaluation point were not standardized. Fourth, we could not compare with the group that underwent surgical intervention. Overall, the results of the study showed the efficacy of conservative treatments with a significant increase in mouth opening, decrease in muscle and/or joint pain, and remission of subjective symptoms. However, it should be considered that some patients with persistent capsular pain may benefit from surgical intervention, including arthrocentesis. Prospective research is needed to investigate the effectiveness of conservative treatments using more objective assessment tools in patients with severe joint damage compared with surgical approaches in a large number of patients.

## Conclusions

This study showed the efficacy of comprehensive conservative treatments in patients with TMJ retrodiscal layer rupture and/or disc perforation. The mean distance of mouth opening was increased, masticatory muscle pain was decreased, and the overall subjective symptoms were decreased. Surgical interventions need to be considered in patients with persistent capsular pain. It is advisable to provide comprehensive conservative treatments first in these patients before applying surgical approach.

## Electronic supplementary material

Below is the link to the electronic supplementary material.

Supplementary Material 1

## Data Availability

Data are available from the corresponding author upon reasonable request.
