# Supplementary material for: Outcome of conservative treatments in patients with TMJ retrodiscal layer rupture or disc perforation
Source: Clin Oral Investig. 2025 Jan 30;29(2):98. doi: 10.1007/s00784-025-06177-9 (PMC11782455; doi:10.1007/s00784-025-06177-9)
Supplement: Supplementary file 1 — Supplementary Material 1 [file 784_2025_6177_MOESM1_ESM.docx]

**Supplementary Table 1** Demographic and clinical characteristics of patients with TMJ retrodiscal layer rupture and/or disc perforation

| Patient No. | Age  (years) | Sex  (M/F) | Systemic diseases | Trauma history | Presence of DJD | Retrodiscal layer rupture/Disc perforation based on MRI |
| --- | --- | --- | --- | --- | --- | --- |
| 1 | 78 | M |  | No | Both^a^ | Rt retrodiscal layer rupture, disc perforation  Lt retrodiscal layer rupture |
| 2 | 56 | M |  | Yes (FI) | Lt | Lt retrodiscal layer rupture |
| 3 | 34 | F | Rheumatoid arthritis | No | Both | Lt disc perforation |
| 4 | 47 | F | Rheumatoid arthritis | No | Lt^a^ | Lt disc perforation |
| 5 | 56 | F |  | Yes (FI) | Both | Lt retrodiscal layer rupture |
| 6 | 73 | F |  | No | Both | Lt retrodiscal layer rupture |
| 7 | 66 | F | Breast cancer surgery history | No | Both | Rt retrodiscal layer rupture |
| 8 | 46 | F | Rheumatoid arthritis | No | Both | Both disc perforation |
| 9 | 57 | F |  | No | Both | Lt retrodiscal layer rupture |
| 10 | 48 | F |  | No | Both^a^ | Lt retrodiscal layer rupture |
| 11 | 51 | F |  | No | Both | Lt retrodiscal layer rupture |
| 12 | 41 | F | Mx for thyroid disease | No | Both | Lt retrodiscal layer rupture |
| 13 | 37 | F | Osteoporosis | Yes (FI) | Both | Lt retrodiscal layer rupture |
| 14 | 62 | F | Breast cancer, anxiety disorder, sleep disorder | No | Lt^b^ | Rt retrodiscal layer rupture |
| 15 | 24 | F | Rheumatoid arthritis | No | Lt | Lt disc perforation |
| 16 | 41 | F |  | No | Both | Lt retrodiscal layer rupture |
| 17 | 54 | F | Breast cancer surgery history | No | Both | Both retrodiscal layer rupture |
| 18 | 47 | F | Mx for depression | Yes (FI) | Both | Rt retrodiscal layer rupture |
| 19 | 48 | F | Osteoporosis | No | Both | Lt retrodiscal layer rupture |
| 20 | 20 | F | Orthodontic treatment history | No | Both | Rt retrodiscal layer rupture |
| 21 | 38 | F |  | No | Both | Lt retrodiscal layer rupture |
| 22 | 48 | F |  | No | Both | Rt retrodiscal layer rupture |
| 23 | 70 | F |  | No | Both | Both disc perforation |
| 24 | 51 | F |  | No | Both | Lt retrodiscal layer rupture |
| 25 | 45 | F |  | No | Rt | Rt retrodiscal layer rupture |
| 26 | 73 | F | Arrhythmia | No | Both | Lt retrodiscal layer rupture |
| 27 | 62 | F | Osteoporosis | No | Both | Rt retrodiscal layer rupture |
| 28 | 22 | M |  | No | Both | Lt retrodiscal layer rupture |
| 29 | 48 | F |  | No | Both | Rt retrodiscal layer rupture, disc perforation |
| 30 | 43 | F |  | No | Both | Both retrodiscal layer rupture |
| 31 | 50 | F | Orthodontic treatment history | No | Both | Rt retrodiscal layer rupture |

DJD, degenerative joint disease; FI, facial injury; Lt, left; MRI, magnetic resonance imaging; Mx, medication; Rt, right; TMJ, temporomandibular joint

^a^ CBCT was not taken in three patients (patient #1, 4, and 10). The diagnosis of degenerative joint diseases in three patients was based on plain radiography.

^b^ Degenerative change was not found in the right joint showing retrodiscal layer rupture in this patient (patient #14).

**Supplementary Table 2** Results of clinical evaluation, clinical diagnosis, and treatment outcomes of patients with TMJ retrodiscal layer rupture and/or disc perforation

| Patient No. | TMD diagnosis | Baseline | | | | Types of treatment^a^ | | | After treatment | | | | Treatment outcome^b^ |
| --- | --- | --- | --- | --- | --- | --- | --- | --- | --- | --- | --- | --- | --- |
|  |  | CMO  (mm) | MMO  (mm) | Muscle palpation | Capsule palpation | Mx | Stabilization splint (months) | Follow-up period (months) | CMO  (mm) | MMO  (mm) | Muscle palpation | Capsule palpation |  |
| 1 | MFP  Both DDw/oR  Both OA | 24 | 24 | Both TM | No pain | NSAID | No | 20 | 25 | 27 | No pain | Lt | 2 |
| 2 | Rt DDwR  Lt DDw/oR  Lt OA | 29 | 33 | No pain | No pain | NSAID | Yes (41) | 49 | 38 | 38 | No pain | No pain | 3 |
| 3 | MFP  Rt DDwR  Lt DDw/oR  Both OA | 14 | 18 | Lt MM  Lt TM | Lt | NSAID | Yes (22) | 26 | 43 | 43 | No pain | No pain | 3 |
| 4 | MFP  Both DDw/oR  Lt OA | 26 | 27 | Both MM | Both | No | Yes (24) | 27 | 33 | 33 | No pain | No pain | 2 |
| 5 | MFP  Both DDw/oR  Both OA | 23 | 30 | Both MM  Both TM | Lt | NSAID | Yes (48) | 50 | 40 | 40 | No pain | Lt | 2 |
| 6 | MFP  Rt DDw/oR  Lt DDw/oR  Both OA | 32 | 33 | Lt MM  Lt TM | Lt | NSAID | Yes (45) | 54 | 42 | 42 | No pain | No pain | 2 |
| 7 | MFP  Both DDw/oR  Both OA | 43 | 44 | Rt MM | Rt | NSAID | Yes (6) | 12 | 51 | 51 | No pain | No pain | 2 |
| 8 | MFP  Both DDw/oR  Both OA | 40 | 40 | Lt MM  Rt TM | Rt | No | No | 26 | 39 | 39 | No pain | No pain | 3 |
| 9 | Rt DDwR  Lt DDw/oR  Both OA | 34 | 38 | No pain | No pain | NSAID | Yes (26) | 18 | 54 | 54 | No pain | No pain | 3 |
| 10 | MFP  Both DDw/oR  Both OA | 37 | 37 | Both MM | No pain | No | Yes (5) | 17 | 38 | 38 | No pain | No pain | 3 |
| 11 | MFP  Rt DDwR  Lt DDw/oR  Both OA | 19 | 22 | Both MM | No pain | NSAID | Yes (35) | 39 | 33 | 33 | Lt MM | Lt | 2 |
| 12 | Both DDw/oR  Both OA | 34 | 34 | No pain | No pain | NSAID | Yes (30) | 35 | 32 | 34 | Rt MM | No pain | 2 |
| 13 | MFP  Both DDw/oR  Both OA | 30 | 42 | Both MM  Lt TM | Both | NSAID,  Muscle relaxant | Yes (29) | 33 | 36 | 39 | Rt MM | Rt | 2 |
| 14 | Rt DDw/oR  Lt DDwR  Lt OA | 28 | 28 | No pain | No pain | No | Yes (4) | 19 | 37 | 37 | No pain | No pain | 3 |
| 15 | MFP  Rt DDwR  Lt DDw/oR  Lt OA | 30 | 35 | Both MM | Lt | NSAID, Muscle relaxant | Yes (23) | 26 | 28 | 31 | Both MM | Both | 2 |
| 16 | MFP  Both DDw/oR  Both OA | 33 | 38 | Both MM  Both TM | No pain | NSAID | Yes (21) | 25 | 34 | 35 | Both MM  Both TM | Both | 2 |
| 17 | Both DDw/oR  Both OA | 43 | 43 | No pain | Lt | NSAID | Yes (10) | 14 | 48 | 50 | No pain | No pain | 2 |
| 18 | MFP  Lt DDwR  Rt DDw/oR  Both OA | 20 | 35 | Both MM  Both TM | Both | No | Yes (18) | 22 | 40 | 42 | Both MM | Rt | 2 |
| 19 | MFP  Both DDw/oR  Both OA | 34 | 41 | Lt MM | Lt | NSAID | Yes (12) | 16 | 53 | 53 | No pain | No pain | 2 |
| 20 | Both DDw/oR  Both OA | 33 | 33 | No pain | Rt | NSAID | Yes (18) | 22 | 33 | 34 | No pain | No pain | 3 |
| 21 | MFP  Both DDw/oR  Both OA | 25 | 29 | Lt MM  Both TM | Rt | NSAID, Muscle relaxants | Yes (16) | 20 | 43 | 43 | No pain | No pain | 2 |
| 22 | MFP  Both DDw/oR  Both OA | 38 | 38 | Rt MM | Rt | No | Yes (1) | 6 | 40 | 40 | No pain | Rt | 2 |
| 23 | MFP  Both DDw/oR  Both OA | 38 | 38 | Both MM  Both TM | No pain | NSAID | Yes (14) | 24 | 37 | 40 | Lt MM | Both | 2 |
| 24 | Both DDw/oR  Both OA | 40 | 40 | No pain | No pain | No | Yes (14) | 19 | 44 | 44 | No pain | No pain | 2 |
| 25 | Rt DDw/oR  Lt DDwR  Rt OA | 35 | 35 | No pain | Rt | NSAID | Yes (13) | 19 | 42 | 43 | No pain | No pain | 2 |
| 26 | MFP  Both DDw/oR  Both OA | 28 | 30 | Both MM  Both TM | Both | NSAID | No | 18 | 27 | 27 | Lt MM | Both | 2 |
| 27 | MFP  Both DDw/oR  Both OA | 25 | 26 | Rt MM  Rt TM | Rt | NSAID | Yes (14) | 17 | 32 | 33 | No pain | No pain | 2 |
| 28 | Both DDw/oR  Both OA | 50 | 50 | No pain | No pain | No | Yes (7) | 18 | 50 | 50 | No pain | No pain | 2 |
| 29 | Both DDw/oR  Both OA | 35 | 38 | No pain | No pain | NSAID | No | 26 | 47 | 47 | No pain | No pain | 3 |
| 30 | Both DDw/oR  Both OA | 32 | 34 | No pain | Rt | No | Yes (6) | 16 | 33 | 34 | No pain | Rt | 2 |
| 31 | MFP  Both DDw/oR  Both OA | 46 | 46 | Both MM | No pain | No | Yes (4) | 19 | 46 | 46 | Both MM | No pain | 2 |

CMO, interincisal distance of comfortable mouth opening; DDwR: disc displacement with reduction; DDw/oR: disc displacement without reduction; MFP, myofascial pain; MM, masseter muscle; MMO, interincisal distance of maximum mouth opening; Mx, medication; NSAID, nonsteroidal anti-inflammatory drug; OA, osteoarthritis or osteoarthrosis; TM, temporalis muscle; TMD, temporomandibular disorders; TMJ, temporomandibular joint

^a^ All patients received physical therapy.

^b^ 1, No improvement or worsening of symptoms; 2, Partial improvement of symptoms; 3, Complete improvement of symptoms

**Supplementary Table 3** Treatment outcomes in patients not wearing oral stabilization splint (*n* = 4) among patients with TMJ retrodiscal layer rupture and/or disc perforation; Mean ± SD, n (%)

| Parameters | Baseline | After treatment | *P* value |
| --- | --- | --- | --- |
| CMO (mm) | 31.8 ± 7.1 | 34.5 ± 10.4 | 0.705 |
| MMO (mm) | 33.0 ± 7.4 | 35.0 ± 9.8 | 0.581 |
| Muscle pain |  |  |  |
| With pain | 3 (75.0) | 1 (25.0) | 0.486 |
| Without pain | 1 (25.0) | 3 (75.0) |  |
| Capsular pain |  |  |  |
| With pain | 2 (50.0) | 2 (50.0) | 1 |
| Without pain | 2 (50.0) | 2 (50.0) |  |
| Treatment outcome |  |  |  |
| No improvement or worsening of symptoms |  | 0 |  |
| Partial improvement of symptoms |  | 2 (50.0) |  |
| Complete improvement of symptoms |  | 2 (50.0) |  |

CMO, interincisal distance of comfortable mouth opening; MMO, interincisal distance of maximum mouth opening; TMJ, temporomandibular joint

Wilcoxon signed rank test and Fisher’s exact test was used to analyze differences in the parameters between the baseline and after treatment.
